# How Are Different Perfectionism Traits Related to Mental Health in Students?

**DOI:** 10.3390/bs14030187

**Published:** 2024-02-27

**Authors:** Paweł Larionow

**Affiliations:** Faculty of Psychology, Kazimierz Wielki University, 85-064 Bydgoszcz, Poland; pavel@ukw.edu.pl

**Keywords:** concern over mistakes, doubts about actions, Frost Multidimensional Perfectionism Scale, parental criticism, parental expectations, perfectionism, personal standards, stress, students, well-being

## Abstract

Multidimensional models of perfectionism postulate the existence of various perfectionism traits, with different effects on mental health. In order to suggest parsimonious targets in psychological interventions for university students, this study aimed to explore *whether*, *how*, and *which* individual perfectionism traits are uniquely associated with stress and well-being. The participants were 253 students aged 18–30 who completed the Frost Multidimensional Perfectionism Scale, the Perceived Stress Scale, and the Warwick–Edinburgh Mental Well-being Scale. Controlling for the common variance of perfectionism traits in statistical analysis, it was shown that (1) Personal Standards were associated with higher well-being and lower stress, (2) Concern over Mistakes and Doubts about Actions were related to lower well-being and higher stress, (3) Parental Expectations and Parental Criticism were not correlated with stress, and (4) Parental Criticism was associated with lower well-being. In the multi-predictor mediation model, with five perfectionism traits as predictors, perceived stress was a significant mediator between several perfectionism traits (i.e., Personal Standards, Concern over Mistakes, and Doubts about Actions) and well-being. Overall, Personal Standards, Concern over Mistakes, and Doubts about Actions seem to be parsimonious psychological targets, with Personal Standards expressing mental health-promoting effects, whereas Parental Expectations and Parental Criticism seem to be less important psychological targets.

## 1. Introduction

Perfectionism is considered a multidimensional personality trait involving perfectionistic strivings (i.e., setting high standards) and perfectionistic concerns (i.e., concern about mistakes and actions as well as subjective perceptions of negative expectations and assessments by others) [1]. Researchers of perfectionism highlighted several categories of factors that contribute to the development of perfectionism, including genetic determinants, personality traits, family factors, and educational and socio–cultural factors (for review, see Kwarcińska et al. [2]), which were reflected in the theory and practice of perfectionism, including the development of psychometric measures of perfectionism.

For assessing perfectionism, Frost et al. [3] developed the 35-item Frost Multidimensional Perfectionism Scale (FMPS), consisting of six perfectionism dimensions, including (1) Concern over Mistakes (e.g., *“I should be upset if I make a mistake”*), (2) Doubts about Actions (e.g., *“Even when I do something very carefully, I often feel that it is not quite right”*), (3) Parental Expectations (e.g., *“My parents set very high standards for me”*), (4) Parental Criticism (e.g., *“My parents never tried to understand my mistakes”*), (5) Personal Standards (e.g., *“It is important to me that I am thoroughly competent in everything I do”*), and (6) Organization (e.g., *“Organization is very important to me”*). Further studies have shown that the last perfectionism trait, the Organization dimension, should be considered as a correlate of perfectionism and not as a dimension of the perfectionism construct [1,4]. Also, research has indicated that Concern over Mistakes, Doubts about Actions, Parental Expectations, and Parental Criticism can be treated as perfectionistic concerns, whereas Personal Standards are an indicator of perfectionistic strivings [1,5]. Some psychometric works on the FMPS have stressed that Parental Expectations with Parental Criticism and Concern over Mistakes with Doubts about Actions could be combined into two factors [6]. In contrast, current psychometric studies have contributed to the five-factor model of the FMPS, indicating its specific correlational patterns with other correlates of perfectionism [4]; therefore, assessing all dimensions of perfectionism seems to be beneficial.

Regarding the impact of perfectionism traits on health, perfectionistic concerns were in general related to poorer mental and somatic health, whereas perfectionistic strivings were associated with better health (for review, see Kwarcińska et al. [2]). In the meta-analysis by Limburg et al. [7], it was suggested that perfectionism can be considered a transdiagnostic risk factor for psychopathologies, including anxiety, depression, and obsessive-compulsive disorders, with relatively strong positive links between these disorders and perfectionistic concerns, as well as with positive but trivial or non-significant links between these disorders and perfectionistic strivings. These conclusions were supported by Lunn et al.’s meta-analysis [8], where the significant positive association between perfectionistic concerns and psychopathology symptoms in young people was noted, whereas the correlation between perfectionistic strivings and these symptoms was smaller. Piotrowski and Bojanowska [4] suggested controlling for the common variance of perfectionism traits in statistical analysis. Such an approach provides more comprehensive and relevant results on the unique role of individual perfectionism dimensions associated with other variables. Therefore, they showed that in multiple regression analyses with five perfectionism traits (as predictors), the Personal Standards dimension was negatively associated with ill-being indicators (i.e., negative emotions, ruminations) while being positively associated with these indicators in bivariate correlations. This indicates that the Personal Standards subscale plays a more adaptive role than other perfectionism dimensions, which were associated positively with the investigated ill-being indicators.

Considering the role of perfectionism in an educational context, recent meta-analyses have supported that parental expectations and parental criticism have increased within the last three decades [9], highlighting the rising role of perfectionism among students. The theoretical and empirical support of a link between perfectionism and stress was presented in past works (e.g., Achtziger and Bayer [10], Suh et al. [11]), including studies among university students [12]. For example, Gil et al. [12] noted a significant positive correlation between perfectionism (a total score of perfectionistic concerns and perfectionistic strivings) and academic stress, with worry and rumination acting as mediators in the association between academic stress and symptoms of anxiety and depression. Garratt-Reed et al. [13] revealed that perfectionistic concerns were associated (in particular, through repetitive negative thinking) with higher levels of academic burnout, whereas perfectionistic strivings were associated with lower levels of academic burnout. Wang and Wu [14] showed that maladaptive perfectionism (a total score of Concern over Mistakes, Parental Expectations, and Doubts about Actions subscales) was associated with lower life satisfaction, and this relationship was mediated via academic burnout among students. It seems that perfectionistic concerns lead to higher levels of ill-being, and this in turn contributes to lower general levels of well-being.

As many previous studies mainly analyzed the links between two composite perfectionism dimensions (i.e., perfectionistic concerns and perfectionistic strivings) and other psychological variables, chiefly ill-being indicators (e.g., psychopathology symptoms), it is unclear (1) *whether*, *how*, and *which* individual perfectionism traits are uniquely associated with well-being indicators as well as with ill-being indicators, and (2) *whether* stress acts as a mediator in the association between individual perfectionism traits and well-being. This study aimed to address these two research questions with mediation analysis. In order to understand the specific role of individual perfectionistic traits (i.e., Concern over Mistakes, Doubts about Actions, Parental Expectations, Parental Criticism, and Personal Standards), all these traits were treated as independent variables, whereas stress was a mediator and well-being was a dependent variable in a mediation model with multiple predictors. The suggestion that controlling for the common variance of perfectionism traits in the statistical analysis approach is beneficial was indicated in previous reports by Piotrowski and Bojanowska [4]. Hence, this statistical approach expressed in a mediation model with multiple predictors was also applied in this study. Additionally, not only ill-being indicators (i.e., stress) but also positive psychological mental health indicators (i.e., well-being) were taken into account. Based on Karademas’s [15] ideas about common and specific predictors of well-being and ill-being, this study aimed to explore whether and which perfectionism traits are uniquely related to either well-being or stress, indicating their specific predictive role, or whether they are related to both of them, indicating their common and simultaneous roles in well-being promotion and ill-being protection. This may help in the optimization of psychological interventions in the higher education context, suggesting parsimonious and effective psychological targets [16].

The aim of this study was (1) to explore and clarify associations of perfectionism traits with stress and well-being, and (2) to examine the mediation role of stress in the relationship between perfectionism traits and well-being among young people. Based on past studies on Frost et al.’s multidimensional model of perfectionism [1,14] as well as on the Polish validation study of the FMPS [4], I expected that the Personal Standards subscale would be associated with lower stress and higher well-being, whereas other perfectionism dimensions (i.e., Concern over Mistakes, Doubts about Actions, Parental Expectations, and Parental Criticism) would be associated with higher stress and lower well-being. I also predicted that stress would be a statistically significant mediator in the relationship between perfectionism dimensions and well-being (in a mediation model with multiple predictors).

## 2. Materials and Methods

### 2.1. Procedure

This study was conducted according to the Declaration of Helsinki Ethical Principles and the approval of the institutional review board of the Faculty of Psychology of Kazimierz Wielki University (No. 2/12 January 2021). This study was anonymous and voluntary and used a paper-and-pencil format. Participants (students) were recruited at Kazimierz Wielki University during classes. All the students provided written informed consent and then filled out a demographic questionnaire and a short battery of self-report questionnaires on perfectionism, stress, and well-being.

### 2.2. Participants

The participants were 253 social science students (207 females and 46 males) at the Kazimierz Wielki University (Bydgoszcz, Poland). The participants’ ages ranged from 18 to 30 years, with a mean age of 20.74 years (*SD* = 2.18). A total of 227 (89.72%) students obtained secondary education, and 26 (10.28%) students had a higher education degree. Among the participants, 131 (51.78%) were single and 122 (48.22%) were in relationships. In terms of residence, 97 (38.34%) lived in villages, 37 (14.62%) in small towns (up to 20,000 inhabitants), 31 (12.25%) in medium-sized towns (from 20,000 to 100,000), and 88 (34.78%) in large cities (above 100,000). Among the respondents, 176 (69.57%) were just studying, and 77 (30.43%) combined studying and working.

### 2.3. Measures

1. The *Frost Multidimensional Perfectionism Scale* (FMPS) [3] was used for assessing perfectionism. The Polish version of the FMPS [4] has 29 items and five subscales: (1) Concern over Mistakes (nine items, e.g., *“I should be upset if I make a mistake”*), (2) Doubts about Actions (four items, e.g., *“Even when I do something very carefully, I often feel that it is not quite right”*), (3) Parental Expectations (five items, e.g., *“My parents set very high standards for me”*), (4) Parental Criticism (four items, e.g., *“My parents never tried to understand my mistakes”*), and (5) Personal Standards (seven items, e.g., *“It is important to me that I am thoroughly competent in everything I do”*). The FMPS uses a 5-point response scale from 1 (*“strongly disagree”*) to 5 (*“strongly agree”*). Higher scores indicate higher levels of perfectionism dimensions.
2. The *Perceived Stress Scale* (PSS-10) [17] was used for assessing perceived stress. The Polish version of the PSS-10 [18] has 10 items (e.g., *“In the last month, how often have you been upset because of something that happened unexpectedly?”*), with a 5-point response scale from 0 (*“never”*) to 4 (*“very often”*). Higher scores indicate higher levels of perceived stress.
3. The *Warwick–Edinburgh Mental Well-being Scale* (WEMWBS) [19] was used for assessing well-being. The Polish version of the WEMWBS [20] has 14 items (e.g., *“I’ve been feeling optimistic about the future”*), with a 5-point response scale from 1 (*“none of the time”*) to 5 (*“all of the time”*). Higher scores indicate higher levels of well-being.

### 2.4. Statistical Analysis

*Jamovi Desktop* v. 2.4.11 was used for all statistical analyses. Descriptive statistics and Pearson correlations between the study variables were calculated. A mediation analysis with multiple predictors was applied. Estimation used the bootstrap percentiles method with 5000 samples and 95% confidence intervals (95% CI). In this mediation analysis, five perfectionism traits were the independent variables, stress was the mediator, and well-being was the dependent variable.

## 3. Results

Descriptive statistics and internal consistency reliability for the study variables are presented in Table 1. All measures had acceptable to good internal consistency reliability, with Cronbach’s alpha from 0.69 to 0.91.

Table 2 presents Pearson correlations between the variables. All perfectionism traits were positively interrelated (*r* from 0.23 to 0.77, *p* < 0.001). All perfectionism traits (except Personal Standards) were positively associated with stress (*r* from 0.24 to 0.54, *p* < 0.001) and negatively with well-being (*r* from −0.33 to −0.52, *p* < 0.001).

The mediation analysis results are presented in Table 3.

Out of five perfectionism traits acting as the predictors in the multiple regression analysis, four were statistically significant predictors of well-being (see Table 3, the total effects, which represent the results of the multiple regression without the mediator in the model; *F*(5,247) = 30.61, *p* < 0.001, R^2^ = 0.38, adjusted R^2^ = 0.37). Personal Standards (beta = 0.21) were a statistically significant positive predictor of well-being, whereas Concern over Mistakes (beta = −0.39), Doubts about Actions (beta = −0.20), and Parental Criticism (beta = −0.26) were statistically significant negative predictors of well-being. Controlling for other perfectionism traits, Parental Expectations were not a statistically significant predictor of well-being (beta = 0.09, *p* = 0.270).

As for links with stress, controlling for other perfectionism traits, Parental Expectations (beta = −0.16) and Parental Criticism (beta = 0.12) were not statistically significantly associated with stress. Conversely, Concern over Mistakes (beta = 0.40) and Doubts about Actions (beta = 0.31) were statistically significantly positively associated with stress, whereas Personal Standards (beta = −0.11) were statistically significantly negatively associated with it (see Table 3, the component; *F*(5,247) = 29.16, *p* < 0.001, R^2^ = 0.37, adjusted R^2^ = 0.36).

Controlling for stress (see Table 3, the direct effects, which represent the results of the multiple regression with the mediator in the model; *F*(6,246) = 56.94, *p* < 0.001, R^2^ = 0.58, adjusted R^2^ = 0.57), the Personal Standards (beta = 0.15) dimension was a statistically significant positive predictor of well-being, whereas Concern over Mistakes (beta = −0.17) and Parental Criticism (beta = −0.19) were statistically significant negative predictors of well-being. Controlling for stress, Doubts about Actions (beta = −0.03, *p* = 0.675) and Parental Expectations (beta = 0.00, *p* = 1.000) were not statistically significant predictors of well-being.

The mediation analysis revealed that there were three statistically significant indirect effects (paths) in the association between perfectionism traits and well-being via stress as the mediator (see Table 3, the indirect effects). The Personal Standards subscale was associated with higher levels of well-being via decreasing stress levels (*p* = 0.045), whereas Concern over Mistakes (*p* < 0.001) and Doubts about Actions (*p* < 0.001) were associated with lower levels of well-being via increased stress levels. Stress did not mediate the relationship between Parental Expectations and well-being (*p* = 0.055) or between Parental Criticism and well-being (*p* = 0.140).

## 4. Discussion

This brief report study aimed to explore and clarify unique associations of perfectionism traits with stress and well-being, and to examine the mediation role of stress in the relationship between perfectionism traits and well-being among students. Overall, the results supported the utility of Frost et al.’s multidimensional five-factor model of perfectionism [3,4].

### 4.1. The Relationship between Perfectionism Traits and Stress, and Well-Being

In bivariate correlational analysis, all perfectionism traits were positively interrelated, which is in line with previous studies [3,4,21]. The majority of perfectionism traits were positively associated with stress and negatively with well-being, except the Personal Standards subscale, which was not associated with stress and well-being. In a more beneficial statistical approach when controlling for the common variance of perfectionism traits in the regression analysis, Personal Standards (beta = 0.21) were a statistically significant positive predictor of well-being, whereas Concern over Mistakes (beta = −0.39), Doubts about Actions (beta = −0.20), and Parental Criticism (beta = −0.26) were statistically significant negative predictors of well-being, with Concern over Mistakes being the strongest predictor. In contrast, Parental Expectations (beta = 0.09) were not a statistically significant predictor of well-being.

As for links with stress (controlling for other perfectionism traits), Parental Expectations and Parental Criticism were not statistically significantly associated with stress, whereas other perfectionism traits were statistically significantly associated with it. In a more comprehensive regression model with all perfectionism traits and controlling for stress, Personal Standards (beta = 0.15) remained a statistically significant positive predictor of well-being, whereas Concern over Mistakes (beta = −0.17) and Parental Criticism (beta = −0.19) remained statistically significant negative predictors of well-being. Doubts about Actions and Parental Expectations were considered statistically insignificant predictors of well-being. Overall, these results indicated that Parental Criticism and Concern over Mistakes were the most impactful perfectionism traits associated with a decrease in well-being, whereas Personal Standards seemed to be associated with an increase in well-being. Given that previous studies have shown that maladaptive perfectionism traits (i.e., Concern over Mistakes, Doubts about Actions, Parental Expectations, and Parental Criticism) were positively related to depression, whereas Personal Standards were negatively related to it [22], these maladaptive perfectionism traits could lead to adverse effects for mental health and decrease well-being via increasing depression levels. Previous reports also indicated that perfectionistic concerns were associated with higher academic burnout partially via repetitive negative thinking, whereas perfectionistic strivings were directly associated with lower academic burnout [13]. In summation, this current study supported the conclusions presented in past work regarding the negative role of perfectionistic concerns for mental health in students and provided new insights on the positive role of the Personal Standards dimension, which was associated with lower stress and higher well-being in this dataset.

In this study, control for the common variance of perfectionism traits in statistical analyses (suggested by Piotrowski and Bojanowska [4]) made it possible to reveal the specific role of individual perfectionism traits in well-being and ill-being. Specifically, the positive role of Personal Standards was shown in regression models, whereas in a bivariate correlational analysis, the Personal Standards scores were not correlated with well-being and ill-being. Therefore, the common variance of perfectionism traits is recommended to be controlled in statistical analysis in order to clarify individual associations of perfectionism traits with other correlates.

### 4.2. The Relationship between Perfectionism Traits and Well-Being via Stress

Considering the mediation analysis results, stress statistically significantly mediated the relationships between three perfectionism traits and well-being. Concern over Mistakes and Doubts about Actions were associated with higher stress, which consecutively enhanced lower well-being. In contrast, Personal Standards were associated with lower stress, which sequentially enhanced higher well-being. These results are in line with Wang and Wu’s report [14], which showed that maladaptive perfectionism traits (expressed in a composite score of Concern over Mistakes, Parental Expectations, and Doubts about Actions subscales) were associated with lower life satisfaction via higher academic burnout. Overall, as maladaptive perfectionism traits were associated with higher perceived stress, their negative role in mental and somatic health could be expressed through enhancing the physiological activation resulting from the development of chronic stress [23]. Thus, the assessment of maladaptive perfectionism traits, accompanied by stress, could be beneficial when providing psychological interventions for students with high levels of maladaptive perfectionism traits.

The mediation analysis results also indicated that Parental Expectations and Parental Criticism were not indirectly associated with well-being via stress. It seems that these two perfectionism traits are associated with lower well-being not through increasing the perceived stress levels but through other psychological mechanisms (for example, see van Houtum et al. [24]). In this study, a relatively homogeneous group of students aged 18–30 was recruited; therefore, in this group of young adults, the role of Parental Expectations and Parental Criticism could be less important than in younger people (or adolescents). Hence, more diverse samples would be beneficial in future work.

### 4.3. Practical Implications of the Study

From a practical point of view, the mediation analysis results indicated that the maladaptive role of Concern over Mistakes and Doubts about Actions could be decreased by reducing perceived stress levels. As perfectionism traits are relatively stable [25,26], psychological interventions could be targeted not only at these traits but also at psychological distress in order to increase well-being in students with these perfectionism traits fairly quickly.

### 4.4. Limitations of the Study

In this cross-sectional study, the mediation model was justified based on past work and tested in a series of statistical analyses. However, longitudinal data would be more appropriate for mediation models, as the casual inferences of the study variables’ directionality cannot be determined in this cross-sectional study. In the current sample, the number of female participants predominated over male participants, and the sample comprised people aged 18–30; hence, the generalizability of the obtained results is limited. Therefore, a broader student sample and control for the roles of gender and age in statistical analysis would be beneficial in future research.

## 5. Conclusions

In this study, among the five perfectionism traits, common and specific predictors of well-being and ill-being (i.e., stress) were indicated, and a potential mechanism linking perfectionism traits with well-being via stress was examined. This was implemented by controlling for the common variance of perfectionism traits in statistical analysis.

Considering the results of all conducted analyses, Personal Standards were associated with higher well-being and lower stress, whereas Concern over Mistakes and Doubts about Actions were associated with lower well-being and higher stress. Moreover, in the mediation analysis, these three perfectionism traits were associated with well-being via stress. Parental Expectations and Parental Criticism in general were not associated with stress, and Parental Criticism was associated with lower well-being. Therefore, Personal Standards, Concern over Mistakes, and Doubts about Actions seem to be parsimonious psychological targets, whereas Parental Expectations and Parental Criticism seem to be less important targets within psychological interventions.

The results also supported the utility of Frost et al.’s multidimensional five-factor model of perfectionism, with the five perfectionism traits showing specific links with stress and well-being. Therefore, assessing the specific role of individual perfectionism traits seems to be important, chiefly in order to provide effective and parsimonious psychotherapeutic approaches for people struggling with high levels of maladaptive perfectionism traits. This study showed that integrating well-being and ill-being indicators simultaneously allowed for a comprehensive understanding of the specific role of individual perfectionism traits in the mental health outcomes of students. Hence, this approach seems advantageous for the theory and practice of perfectionism.

## Figures and Tables

**Table 1:** Descriptive statistics and internal consistency reliability for the variables (*n* = 253).

	Personal Standards	Concern over Mistakes	Doubts about Actions	Parental Expectations	Parental Criticism	Stress	Well-Being
Mean	22.81	26.74	11.99	13.14	9.17	22.41	45.09
Standard deviation	5.88	8.94	3.61	5.79	4.49	6.84	9.46
Minimum	8	9	4	5	4	6	18
Maximum	35	45	20	25	20	39	69
Skewness	−0.21	0.01	0.06	0.37	0.78	0.04	−0.03
Kurtosis	−0.27	−0.73	−0.38	−0.94	−0.28	−0.11	−0.06
Cronbach’s alpha	0.82	0.91	0.69	0.89	0.85	0.87	0.91

**Table 2:** Pearson correlations between the variables (*n* = 253).

	Personal Standards	Concern over Mistakes	Doubts about Actions	Parental Expectations	Parental Criticism	Stress	Well-Being
Personal Standards	—						
Concern over Mistakes	0.43 ***	—					
Doubts about Actions	0.23 ***	0.67 ***	—				
Parental Expectations	0.26 ***	0.46 ***	0.48 ***	—			
Parental Criticism	0.23 ***	0.50 ***	0.53 ***	0.77 ***	—		
Stress	0.12	0.54 ***	0.54 ***	0.24 ***	0.34 ***	—	
Well-being	−0.04	−0.52 ***	−0.51 ***	−0.33 ***	−0.44 ***	−0.71 ***	—

*Note*. *** *p* < 0.001.

**Table 3:** Mediation analysis results with stress as the mediator (*n* = 253).

Type	Effect	Estimate	SE	95% CI		Beta	z	*p*
				Lower	Upper			
Indirect	Personal Standards ⇒ Stress ⇒ Well-being	0.1	0.05	−0.02	0.24	0.06	2	0.045
	Concern over Mistakes ⇒ Stress ⇒ Well-being	−0.24	0.05	−0.35	−0.13	−0.22	−4.79	<0.001
	Doubts about Actions ⇒ Stress ⇒ Well-being	−0.46	0.11	−0.69	−0.23	−0.17	−4.06	<0.001
	Parental Expectations ⇒ Stress ⇒ Well-being	0.14	0.07	−0.01	0.31	0.09	1.92	0.055
	Parental Criticism ⇒ Stress ⇒ Well-being	−0.15	0.1	−0.38	0.06	−0.07	−1.48	0.14
Component	Personal Standards ⇒ Stress	−0.13	0.07	−0.30	0.03	−0.11	−2.03	0.042
	Stress ⇒ Well-being	−0.78	0.07	−0.93	−0.63	−0.56	−10.96	<0.001
	Concern over Mistakes ⇒ Stress	0.3	0.06	0.18	0.42	0.4	5.33	<0.001
	Doubts about Actions ⇒ Stress	0.59	0.13	0.3	0.87	0.31	4.37	<0.001
	Parental Expectations ⇒ Stress	−0.18	0.09	−0.39	0.02	−0.16	−1.95	0.051
	Parental Criticism ⇒ Stress	0.19	0.13	−0.08	0.47	0.12	1.49	0.136
Direct	Personal Standards ⇒ Well-being	0.23	0.07	0.09	0.38	0.15	3.17	0.002
	Concern over Mistakes ⇒ Well-being	−0.18	0.07	−0.32	−0.05	−0.17	−2.69	0.007
	Doubts about Actions ⇒ Well-being	−0.07	0.16	−0.41	0.25	−0.03	−0.42	0.675
	Parental Expectations ⇒ Well-being	0	0.11	−0.24	0.25	0	0	1
	Parental Criticism ⇒ Well-being	−0.40	0.14	−0.72	−0.09	−0.19	−2.81	0.005
Total	Personal Standards ⇒ Well-being	0.34	0.09	0.16	0.53	0.21	3.78	<0.001
	Concern over Mistakes ⇒ Well-being	−0.42	0.08	−0.58	−0.25	−0.39	−5.35	<0.001
	Doubts about Actions ⇒ Well-being	−0.52	0.18	−0.96	−0.12	−0.20	−2.83	0.005
	Parental Expectations ⇒ Well-being	0.14	0.13	−0.17	0.46	0.09	1.1	0.27
	Parental Criticism ⇒ Well-being	−0.54	0.17	−0.93	−0.14	−0.26	−3.16	0.002

*Note*. Betas are completely standardized effect sizes.

## Data Availability

The raw data supporting the conclusions of this article will be made available by the author upon reasonable request.
